# The physiological roles of vesicular GABA transporter during embryonic development: a study using knockout mice

**DOI:** 10.1186/1756-6606-3-40

**Published:** 2010-12-30

**Authors:** Kenzi Saito, Toshikazu Kakizaki, Ryotaro Hayashi, Hiroshi Nishimaru, Tomonori Furukawa, Yoichi Nakazato, Shigeo Takamori, Satoe Ebihara, Masakazu Uematsu, Masayoshi Mishina, Jun-ichi Miyazaki, Minesuke Yokoyama, Shiro Konishi, Koichi Inoue, Atsuo Fukuda, Manabu Fukumoto, Kenji Nakamura, Kunihiko Obata, Yuchio Yanagawa

**Affiliations:** 1 Department of Genetic and Behavioral Neuroscience, Gunma University Graduate School of Medicine, Maebashi 371-8511, Japan; 2 The Graduate University for Advanced Studies, Hayama, Kanagawa 240-0193, Japan; 3 Japan Science and Technology Agency, CREST, Tokyo 102-0075, Japan; 4 Department of Pathology, Institute of Development, Aging and Cancer, Tohoku University, Sendai 980-8575, Japan; 5 Graduate School of Comprehensive Human Science, University of Tsukuba, Tsukuba 305-8577, Japan; 6 Department of Physiology, Hamamatsu University School of Medicine, Hamamatsu 431-3192, Japan; 7 Department of Human Pathology, Gunma University Graduate School of Medicine, Maebashi 371-8511, Japan; 8 Doshisha University Faculty of Life and Medical Sciences, Kyotanabe, Kyoto 610-0394, Japan; 9 Department of Cellular Neurobiology, Graduate School of Medicine, University of Tokyo, Tokyo 113-0033, Japan; 10 Department of Materials Science, Toyohashi University of Technology, Toyohashi 441-8580, Japan; 11 Department of Molecular Neurobiology and Pharmacology, Graduate School of Medicine, University of Tokyo, Tokyo 113-0033, Japan; 12 Division of Stem Cell Regulation Research, G6, Osaka University Graduate School of Medicine, 2-2 Yamadaoka, Suita, Osaka, 565-0871, Japan; 13 Mitsubishi Kagaku Institute of Life Sciences, MITILS, 11 Minamiooya, Machida, Tokyo, 194-8511, Japan; 14 Obata Research Unit, RIKEN Brain Science Institute, Wako 351-0198, Japan

## Abstract

**Background:**

The vesicular GABA transporter (VGAT) loads GABA and glycine from the neuronal cytoplasm into synaptic vesicles. To address functional importance of VGAT during embryonic development, we generated global VGAT knockout mice and analyzed them.

**Results:**

VGAT knockouts at embryonic day (E) 18.5 exhibited substantial increases in overall GABA and glycine, but not glutamate, contents in the forebrain. Electrophysiological recordings from E17.5-18.5 spinal cord motoneurons demonstrated that VGAT knockouts presented no spontaneous inhibitory postsynaptic currents mediated by GABA and glycine. Histological examination of E18.5 knockout fetuses revealed reductions in the trapezius muscle, hepatic congestion and little alveolar spaces in the lung, indicating that the development of skeletal muscle, liver and lung in these mice was severely affected.

**Conclusion:**

VGAT is fundamental for the GABA- and/or glycine-mediated transmission that supports embryonic development. VGAT knockout mice will be useful for further investigating the roles of VGAT in normal physiology and pathophysiologic processes.

## Background

GABAergic and glycinergic neurotransmissions play critical roles in the central nervous system (CNS), because they regulate network activity and are essential for a number of brain functions, such as cognition, perception, movement and respiration. In the adult mammalian CNS, GABA and glycine are the main inhibitory neurotransmitters, but in fetal life and early postnatal development, both neurotransmitters act as either excitatory or inhibitory, depending on the intracellular chloride concentration.

GABA is synthesized from glutamic acid by glutamate decarboxylase (GAD) [1] and is accumulated into synaptic vesicles by the vesicular GABA transporter (VGAT) [2,3]. Two isozymes of GAD, GAD65 and GAD67, are primarily expressed in GABAergic neurons [4,5]. GAD65 knockout mice exhibit spontaneous seizures, elevated anxiety and altered sensitivity to pain [6,7]. GAD67 knockout mice die of cleft palate at birth [8]. VGAT is present in both GABAergic and glycinergic neurons and is also called the vesicular inhibitory amino acid transporter (VIAAT) [3,9]. In addition to its presence at GABAergic and glycinergic synapses, the role of VGAT/VIAAT in GABA and glycine release is supported by electrophysiological evidence from primary cultured hippocampal or spinal cord neurons of VGAT knockout mice [10] and VGAT-transfected secretory cells [11]. VGAT knockout mice die perinatally and show a hunched posture, cleft palate and omphalocele [10].

Divergent roles for the VGAT proteins are implicated in the nervous system. However, the contribution of VGAT to tissues or cells outside of the CNS remains largely unclear. For example, neither muscle, lung nor liver phenotypes have been reported for these knockout mice. We independently generated VGAT knockout mice. To further investigate the roles of VGAT during development, we performed histopathological analyses in VGAT knockout muscle, lung and liver at an embryonic stage. These mice showed a reduction in the trapezius muscles, smaller saccules in the lung, and congestion in the liver. In addition, in VGAT knockout spinal cord motoneurons (MNs), spontaneous inhibitory postsynaptic currents (IPSCs) were absent. These experiments indicate that VGAT has an important role in the GABA- and/or glycine-mediated transmission that supports life. Preliminary results have been published in an abstract form [12].

## Results

### Generation of VGAT^-/- ^mice

**Figure 1:**
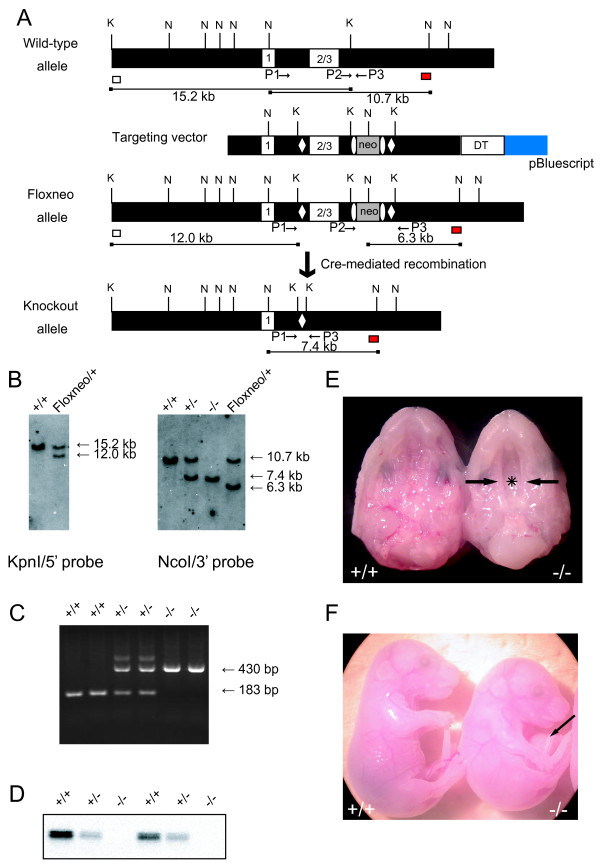
**Generation of VGAT^-/- ^mice**. (A) Schematic representation of the wild-type VGAT allele, the targeting vector, the VGAT-floxneo allele, and the VGAT knockout allele. Exons are represented by numbered white boxes. LoxP sites (open diamonds) and a PGK-Neo cassette (neo; gray box) flanked by the frt sites (open ellipses) were introduced into the wild-type VGAT locus by homologous recombination to produce the floxneo allele. The probes used for Southern blot analysis are indicated as white (5' probe) and red (3' probe) boxes. The expected sizes of the KpnI- and NcoI-digested genomic DNA fragments hybridized with the 5' and 3' probes, respectively, are indicated as lines under the schemes. Relevant restriction sites are indicated as follows: K, KpnI; N, NcoI. PCR primers are indicated as arrows. (B) (Left) Southern blot analysis of KpnI-digested genomic DNA isolated from VGAT^+/+ ^(+/+) and VGAT^floxneo/+ ^(Floxneo/+) mice using the 5' probe indicated in A. The wild-type allele corresponds to the 15.2 kb band, whereas the floxneo allele corresponds to the 12.0 kb band. (Right) Southern blot analysis of NcoI-digested genomic DNA isolated from VGAT^+/+ ^(+/+), VGAT^+/- ^(+/-), VGAT^-/- ^(-/-), and VGAT^floxneo/+ ^(Floxneo/+) mice using the 3' probe indicated in A. The wild-type allele, the knockout allele, and the floxneo allele correspond to the 10.7 kb, 7.4 kb, and 6.3 kb bands, respectively. (C) Genotyping of offspring from intercrosses of VGAT^+/- ^mice by PCR. Three primers were used (see Methods). Primers P2 and P3 produce a 183 bp fragment that represents the wild-type allele, whereas primers P1 and P3 produce a 430 bp fragment that represents the knockout allele. (D) Western blot analysis of E18.5 whole brain homogenates from VGAT^+/+ ^(+/+), VGAT^+/- ^(+/-), VGAT^-/- ^(-/-) using the anti-VGAT antibody directed against an N-terminal epitope. (E) Ventral views of the upper jaw of E18.5 VGAT^+/+ ^(+/+) and VGAT^-/- ^(-/-) mice. In contrast to the completely fused palate of a VGAT^+/+ ^mouse, secondary palatal shelves of a VGAT^-/- ^mouse did not contact each other (arrows), and its nasal cavity (asterisk) could be seen. (F) Lateral views of E18.5 VGAT^+/+ ^(+/+) and VGAT^-/- ^(-/-) mice. An arrow indicates omphalocele in a VGAT^-/- ^mouse. In addition, the VGAT^-/- ^mouse showed an extremely hunched position in contrast to the VGAT^+/+ ^mouse.

The targeting strategy used for the generation of VGAT knockout mice is shown in Figure 1A. Exons 2 and 3 encode the putative ten-transmembrane domain and C-terminus of the VGAT protein [3,13], and accordingly, the deletion of these regions was expected to destroy the function of the VGAT protein. Correctly targeted ES cell clones isolated were microinjected into blastocysts to generate chimeric mice. These mice were then crossed with C57BL/6 mice to generate heterozygous mice carrying one floxneo allele (VGAT^floxneo/+ ^mice).

We generated the VGAT knockout allele by crossing VGAT^floxneo/+ ^mice with CAG-Cre mice, in which Cre recombinase is expressed ubiquitously [14]. Genotyping was performed by Southern blot analysis (Figure 1B) and PCR (Figure 1C), and the DNA sequences around the loxP site in the knockout allele were also confirmed (data not shown). To obtain homozygous VGAT knockout (VGAT^-/-^) mice, we intercrossed the VGAT^+/- ^mice. Western blot analysis revealed no VGAT protein expression in embryonic day (E) 18.5 VGAT^-/- ^brain, whereas VGAT protein expression in VGAT^+/- ^mouse brains was reduced to about half of the wild-type level (Figure 1D). All E18.5 VGAT^-/- ^fetuses displayed cleft palate (Figure 1E) and omphalocele (Figure 1F), phenotypes that are consistent with those described by Wojcik et al. [10].

No VGAT^-/- ^mice survived beyond birth (Table 1). To estimate the time of death of VGAT^-/- ^mice, we performed timed matings of the VGAT^+/- ^mice and obtained the fetuses via cesarean section. Among the E18.5 offspring derived from the intercrosses of VGAT^+/- ^mice, VGAT^-/- ^fetuses were obtained at the expected Mendelian ratio (27.3%, 77 VGAT^-/- ^of 282 littermates) and more than 97% of them (75 of 77) were alive (judged by their umbilical beats or heartbeats, Table 1). When delivered by cesarean section on E18.5, both VGAT^+/+ ^(7 of 7) and VGAT^+/- ^(11 of 12) fetuses began respiration, but none of the VGAT^-/- ^fetuses (n = 7) began to breathe. Therefore, it is probable that VGAT^-/- ^mice died at birth due to respiratory failure.

**Table 1:** Genotypes of offspring from intercrosses of VGAT ^+/-^ mice and phenotypes of VGAT ^-/-^ mice

Age	Genotype			Phenotype		
		
	+/+	+/-	-/-	No. of -/- found dead	No. of -/- with omphalocele	No. of -/- with cleft palate
**E18.5**	69 (24.5%)	136 (48.2%)	77 (27.3%)	2/77*	77/77*	29/29*
						
**Newborn**	22 (19.6%)	76 (67.9%)	14 (12.5%)	14/14*	Not determined	Not determined

*affected/examined

### Elevations in GABA and glycine contents in VGAT^-/- ^forebrains

**Figure 2:**
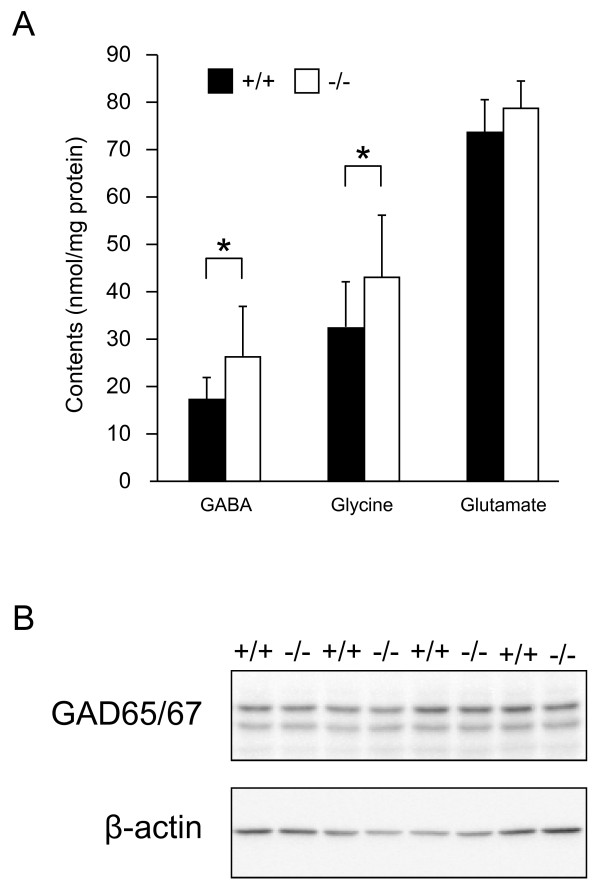
**Neurotransmitter contents and expression levels of GAD65 and GAD67**. (A) Neurotransmitter contents of E18.5 mouse forebrain. VGAT^-/- ^mice showed significantly higher levels of GABA and glycine than VGAT^+/+ ^mice, but not glutamate. Values represent means ± SD (*P < 0.05; Student's t-test, n = 5-13 per group). (B) Western blot analysis. The expression level of GAD65 and GAD67 in whole brain homogenates was not significantly different between VGAT^+/+ ^(+/+) and VGAT^-/- ^(-/-) mice. Equal amounts of protein were loaded and probed with an antibody that recognizes both GAD65 and GAD67. For the statistical comparison, the same blot was probed with anti-β-actin antibody as an internal control and measurements for GAD65 and GAD67 bands were normalized to the β-actin bands (GAD65: VGAT^+/+ ^100 ± 23%; VGAT^-/- ^111 ± 20%, n = 4, P = 0.71) (GAD67: VGAT^+/+ ^100 ± 18%; VGAT^-/- ^93 ± 17%, n = 4, P = 0.80).

In the absence of vesicular storage, neurotransmitter levels can be altered, and this alteration depends on the absence of the vesicular transporter. For example, monoamines are drastically reduced in vesicular monoamine transporter 2 knockout brains [15], but acetylcholine (ACh)  is increased in vesicular acetylcholine transporter (VAChT) knockout brains compared to control wild-type brains [16]. Therefore, we measured the amount of the neurotransmitters, GABA, glycine and glutamate in E18.5 VGAT^-/- ^forebrain by HPLC. As shown in Figure 2A, VGAT^-/- ^fetuses showed significant increases in both GABA and glycine, but not glutamate, compared to VGAT^+/+ ^fetuses. It is possible that the increase in GABA content in VGAT^-/- ^fetuses was due to the elevated expression levels of GABA-synthesizing enzymes. To test for this possibility, we analyzed the expression levels of GAD65 and GAD67 in the embryonic brains. Our Western blot analysis showed that the expression levels of both GAD65 and GAD67 in VGAT^+/+ ^and VGAT^-/- ^brains were similar (Figure 2B). These results indicate that the increase in GABA content was not derived from elevated amounts of GABA-synthesizing enzymes in VGAT^-/- ^embryos.

### Absence of functional inhibitory synaptic transmission in the VGAT^-/- ^spinal cord

**Figure 3:**
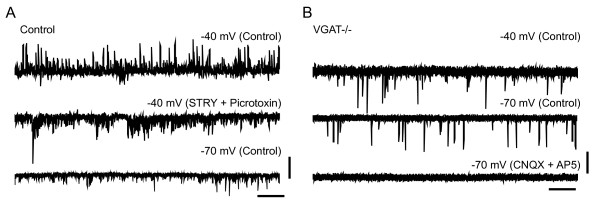
**Electrophysiological characteristics of synaptic transmission in the VGAT^-/- ^spinal cord**. (A) A representative whole-cell patch-clamp recordings from control motoneurons (MNs) in a voltage clamp. In control motoneurons, spontaneous outward currents were observed when the membrane potential was held at -40 mV (upper trace). These currents were blocked by bath application of strychnine (STRY, 0.5 μM) and picrotoxin (50 μM) (middle trace). Inward currents persisted in the presence of these antagonists and became prominent when the membrane potential was held at -70 mV. (B) In VGAT^-/- ^(VGAT-/-) MNs, no spontaneous outward currents were observed (upper trace). Inward currents observed at -70 mV (middle trace) were blocked by CNQX (10 μM) and AP5 (50 μM). Vertical bars indicate 100 pA (A) and 50 pA (B). Horizontal bars indicate 2 s (A, B).

To examine the physiological nature of synaptic inputs to spinal MNs, we performed whole-cell patch-clamp recordings using isolated spinal cord preparations taken from VGAT^-/- ^and control mouse embryos. In these preparations, the neuronal connections within the spinal cord are kept relatively intact [17]. In control lumbar MNs, spontaneous outward currents were observed when the membrane potential was depolarized at -40 mV above the chloride ion reversal potential (approximately -78 mV in the present experimental condition). These currents were blocked by bath application of the glycinergic antagonist strychnine and the GABAergic antagonist picrotoxin, indicating that the currents were IPSCs (n = 8, Figure 3A). In contrast, we did not detect such spontaneous IPSCs in VGAT^-/- ^MNs (n = 12, Figure 3B). When the membrane potential was held at -70 mV, spontaneous inward currents were observed both in control and VGAT^-/- ^MNs (Figure 3A and 3B). These inward currents were abolished by the concomitant bath application of the ionotropic glutamate receptor blockers, a non-NMDA receptor antagonist 6-cyano-7-nitroquinoxaline-2,3-dione (CNQX) and an NMDA receptor antagonist D-2-amino-5-phosphonovaleric acid (AP5), indicating that MNs received excitatory synaptic transmission in the VGAT^-/- ^spinal cord.

### Alterations in body weight, response to stimuli, trapezius muscle, liver and lung of VGAT^-/- ^mice

Wojcik et al. [10] reported that VGAT^-/- ^mice display the phenotypes such as cleft palate, omphalocele, hunched posture, immobility and stiffness. However, we proposed that there would be some other alterations in VGAT^-/- ^mice because VGAT is an essential molecule for GABAergic and glycinergic transmission. To address the question whether VGAT is essential for fetal growth, we initially measured the body weight of VGAT^-/- ^fetuses compared to VGAT^+/+ ^and VGAT^+/- ^littermates. The body weight of the E18.5 VGAT^-/- ^fetuses was significantly lower than that of VGAT^+/+ ^and VGAT^+/- ^fetuses (VGAT^+/+^: 1.18 ± 0.11 grams, n = 17; VGAT^+/-^: 1.20 ± 0.08 grams, n = 45; VGAT^-/-^: 1.05 ± 0.11 grams, n = 32 [mean ± SD], P < 0.001, one-way ANOVA, post hoc Fisher's least significant difference test). These results indicate that VGAT is important for fetal growth.

VGAT^-/- ^fetuses exhibited immobility and lacked spontaneous limb and body movements, which are consistent with a report by Wojcik et al. [10]. To further address the question of whether the lack of movement in VGAT^-/- ^fetuses was restricted to a defect in spontaneous movement, we examined the response of E18.5 VGAT^-/- ^fetuses to mechanical stimuli. VGAT^+/+^, VGAT^+/- ^and VGAT^-/- ^fetuses were obtained via cesarean section and maintained alive in phosphate buffered saline. None of the VGAT^-/- ^fetuses (n = 16) responded to a pinch of the tail by forceps, but all VGAT^+/+ ^(n = 16) and VGAT^+/- ^(n = 53) fetuses responded with a twisting of the trunk. These results suggest that VGAT^-/- ^fetuses suffer from severe impairments in motor function.

**Figure 4:**
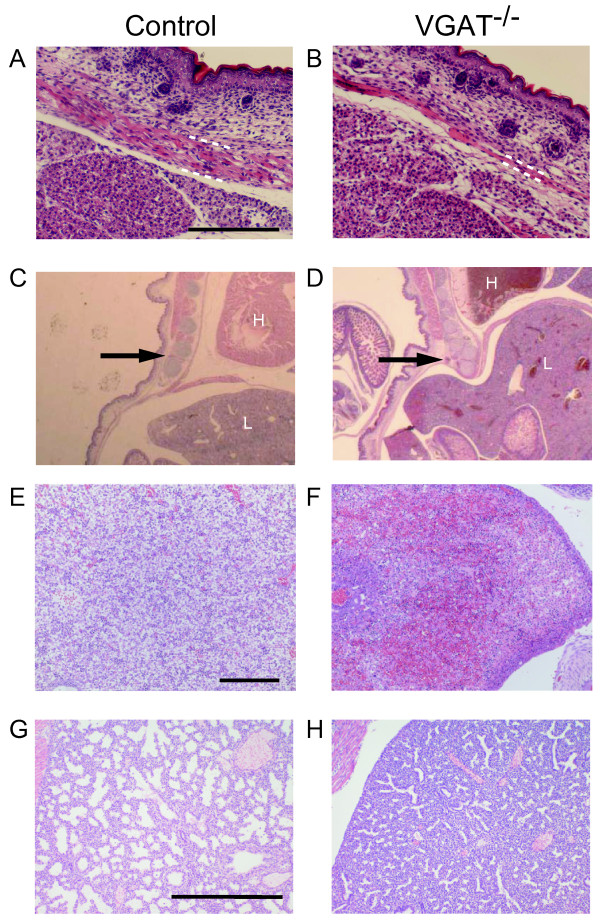
**Histological analysis of VGAT^-/- ^mice**. Histological analysis (hematoxylin and eosin staining) of trapezius muscle (A, B), sagittal sections (C, D), liver (E, F) and lung (G, H) from E18.5 VGAT^-/- ^mice (B, D, F, H) and control mice (A, C, E, G). (A, B) The trapezius muscle (bounded partly by white dashed lines) of VGAT^-/- ^mouse (B) was thinner than the control mouse (A). Scale bar: 200 μm. (C, D) The VGAT^-/- ^ribs (arrow in D) in the lower part were depressed, and their position was inside compared to the control ribs (arrow in C). H, heart; L, liver. (E, F) Red blood cell congestion was characteristic of VGAT^-/- ^liver, but not control liver. Scale bar: 200 μm. (G, H) The VGAT^-/- ^lung contained much less alveolar space than the control lung. Scale bar: 500 μm.

Because the hunched posture observed in VGAT^-/- ^mice (Figure 1F) suggested defects in skeletal muscles or bones [18,19], we performed a histological examination of skeletal muscles and bones in the E18.5 embryos. Trapezius muscle was thinner in VGAT^-/- ^mice than in the control mice (Figure 4A, B). The VGAT^-/- ^ribs in the lower part were depressed, and their position was retracted toward the inside compared to control (Figure 4C, D). Furthermore, the spaces between each rib appeared narrower in VGAT^-/- ^mice compared to the control mice (Figure 4C, D). Abdominal organs were examined histologically in an attempt to identify pathological findings associated with defects in VGAT^-/- ^mice. Not only omphalocele (Figure 1F) but also hepatic congestion were characteristic of VGAT^-/- ^embryos (Figure 4E, F). These results suggest that the crouching posture caused by an imbalance of the strength between the dorsal and ventral muscles made the thorax expand ineffectively, leading to an increase in intra-abdominal pressure in VGAT^-/- ^embryos. Although omphalocele can be caused by a malformation of the ventral body wall [20], the rectus abdominis muscle showed no apparent abnormality in VGAT^-/- ^mice (data not shown).

The VGAT^-/- ^mice lacked autonomous and joggling-induced breathing movements, consistent with the report by Fujii et al. [21]. However, there is still uncertainty regarding the mechanism responsible for the respiratory failure caused by the loss of VGAT protein. To understand the pathology causing respiratory failure in VGAT^-/- ^mice, we fixed embryos at E18.5, the day prior to birth, and performed pathohistology of the lungs. Examination under a microscope revealed that the VGAT^-/- ^lung barely contained alveolar spaces compared to the control lung (Figure 4G, H). A possible cause of atelectasis was reported defect in the diaphragm [22]. We examined the VGAT^-/- ^diaphragm histologically, but we didn't detect any difference in the diaphragm between the VGAT^-/- ^and control mice.

The alterations in the VGAT^-/- ^muscle, liver and lung were likely caused by the loss of VGAT in the CNS, but not the loss of VGAT in the peripheral tissue because VGAT transcripts were detected in brain and spinal cord, but not in muscle, liver or lung [2,3].

### Comparison of cleft palate and omphalocele between VGAT^-/- ^mice and GAD67^-/- ^mice

**Figure 5:**
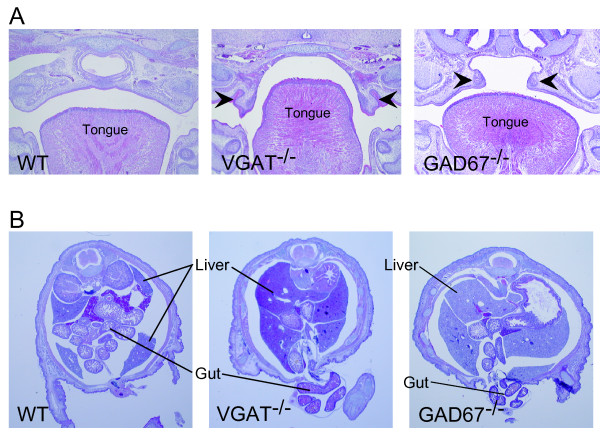
**Histological comparison of omphalocele and cleft palate between VGAT^-/- ^and GAD67^-/- ^mice**. (A) Hematoxylin-eosin stained coronal sections of the facial region of E18.5 wild-type (left panel), VGAT^-/- ^(middle panel) and GAD67^-/- ^(right panel) mice. Histological examination revealed that the secondary palatal shelves (arrowhead) of VGAT^-/- ^mice were directed vertically down along the side of tongue in contrast to the fused palate of wild-type mice. In the cleft palate of GAD67^-/- ^mice (right panel), palatal shelves failed to fuse but were elevated horizontally unlike those in VGAT^-/- ^mice. (B) Hematoxylin-eosin stained coronal sections of the umbilical region of E18.5 wild-type (left panel), VGAT^-/- ^(middle panel) and GAD67^-/- ^(right panel) embryos. The ventral body wall of the VGAT^-/- ^mice did not close and the gut protruded from the peritoneal cavity. In contrast to VGAT^-/- ^mice, the ventral body wall of wild-type mice closed around the umbilicus, and the gut had already returned to the peritoneal cavity. The omphalocele in GAD67^-/- ^mice was less severe than in VGAT^-/- ^mice because a large amount of gut was observed at the umbilical level in VGAT^-/- ^mice compared to GAD67^-/- ^mice.

Cleft palate is exhibited by VGAT^-/- ^and GAD67^-/- ^mice [8,10,23], demonstrating that GABAergic transmissions are involved in palatogenesis. Because VGAT and GAD67 exhibit different molecular functions, we investigated whether the severity of cleft palate was different between VGAT^-/- ^and GAD67^-/- ^mice. Figure 5A shows hematoxylin-eosin staining of coronal sections from the oral region. In the cleft palate of VGAT^-/- ^mice, the palatal shelves remained vertical along the sides of the tongue (3 of 3). However, in GAD67^-/- ^mice, the palatal shelves were elevated up to a horizontal position (3 of 3). In one of the GAD67^-/- ^mice, the palatal shelves even fused with each other completely (data not shown). These observations suggest that palatogenesis progresses further in GAD67^-/- ^mice than in VGAT^-/- ^mice. Our observations also suggest that cleft palate in VGAT^-/- ^mice is more severe than in GAD67^-/- ^mice.

The observation of omphalocele in VGAT^-/- ^mice prompted us to investigate whether GAD67^-/- ^mice displayed omphalocele. We found omphalocele in GAD67^-/- ^and VGAT^-/- ^mice (Figure 5B), indicating that GABA signaling is involved in its onset. The incidence rate of omphalocele in GAD67^-/- ^mice was 43% (17 of 40), whereas the incidence in VGAT^-/- ^mice was 100% (77 of 77; see also Table 1). Thus, the penetrance of omphalocele in GAD67^-/- ^mice was lower than in VGAT^-/- ^mice. The size of omphalocele appeared to be larger in VGAT^-/- ^mice than in GAD67^-/- ^mice. Taken together, these data suggest omphalocele in GAD67^-/- ^mice is less severe than in VGAT^-/- ^mice, similar to what was observed with the cleft palate.

## Discussion

The present work addresses the contribution of VGAT to embryonic development. We generated VGAT mice and found that VGAT is fundamental for GABA and/or glycine release in the spinal cord. Moreover, in the absence of VGAT, there are profound effects on muscle, liver and lung during embryonic development. These observations bear important consequences for understanding the functional roles of VGAT from the cellular to the whole-body level.

### Generation of VGAT knockout mice

Wojcik et al. [10] generated VGAT knockout mice, in which a mutation was inserted into exon 1, and these mice exhibit cleft palate and omphalocele. Here, we generated floxed VGAT knockout mice, in which exons 2 and 3 of the VGAT gene were flanked by loxP sites. Crossing the floxed VGAT mice to CAG-Cre mice reproduced the phenotypes of cleft palate and omphalocele. Exons 1 and 2/3 encode the cytoplasmic domain and the transmembrane domain, respectively [3,13]. Our results demonstrate that exons 2 and 3 are dispensable for the function of VGAT.

VGAT^floxneo/floxneo ^mice were born at the expected frequency, were viable, did not have a cleft palate or omphalocele, and were overtly indistinguishable from their wild-type littermates. Western blot showed that the level of VGAT protein expression in VGAT^floxneo/floxneo ^brain was not different from the wild-type brain (Additional file 1: Supplementary Figure S1). These results suggest that a loxP sequence and an frt-flanked phosphoglycerate kinase promoter-driven neomycin-resistance gene (PGK-Neo) inserted into intron 1 and the 3'-flanking region of the VGAT gene, respectively, do not affect the expression level of VGAT protein. Therefore, the VGAT-floxneo allele allowed for Cre-mediated conditional inactivation of the VGAT gene, and mice carrying these alleles will be useful in examining VGAT function at different developmental stages and in distinct cell types.

### Increase of overall GABA and glycine contents in VGAT^-/- ^forebrains

We showed that both GABA and glycine contents were increased in the VGAT^-/- ^forebrain, but not an excitatory neurotransmitter glutamate, which is transported into synaptic vesicles by vesicular glutamate transporters. An increase in GABA content in VGAT^-/- ^mice is similar to that in ACh contents in VAChT knockout mice [16], but it is opposite of the decrease in monoamine contents in vesicular monoamine transporter 2 knockout mice. In *C. elegans*, the mutational inactivation of VGAT also leads to an increase in GABA immunoreactivity in GABAergic neurons [24]. In VAChT knockout mice the amount of the ACh-synthesizing enzyme choline acetyltransferase (ChAT) is increased at the mRNA and protein levels compared to their wild-type littermates, suggesting that the change in ChAT expression may be related to a compensatory mechanism due to the lack of ACh release [16]. Conversely, the amounts of the GABA-synthesizing enzymes GAD65 and GAD67 were not different in between the brains of VGAT^-/- ^mice and their control littermates (Figure 2B). Therefore, it is possible that the increased GABA in VGAT^-/- ^brain was due to a reduction in their degradation. GABA and glycine are released from presynaptic neurons into the synaptic cleft and are retrieved in neurons and glial cells by plasma membrane transporters [25,26]. GABA and glycine taken up in glial cells are further metabolized, but the GABA and glycine taken up in neurons are directly recycled into synaptic vesicles [27,28]. Because degradation systems for both GABA and glycine are mainly localized to glial cells [27,29], the transport into glial cells from the synaptic cleft is important for their degradation. Because the synaptic release of GABA and glycine was absent in VGAT^-/- ^mice, the deletion of VGAT may result in little or no transport of GABA and glycine into glial cells. GABA and glycine then accumulate in the GABAergic and glycinergic neurons, respectively, but they are not degraded in the glial cells of VGAT^-/- ^mice.

### Contribution of VGAT to motor function

In the embryonic spinal cord of rodents, synaptic transmission to MNs mediated by GABA and glycine is prominent from the early fetal period [30,31]. Our results from the electrophysiological recordings of spinal cord MNs indicate that the inhibitory synaptic transmission was clearly absent in the VGAT knockout MNs, but that the excitatory synaptic transmission was present. Our results also suggest the absence of other functional mechanisms that transport GABA and/or glycine into synaptic vesicles in these synapses. VGAT^-/- ^fetuses at E17.5-18.5 not only were completely immobile and stiff, but also none of them responded to mechanical stimuli by pinching the limb or the tail. Therefore, it is probable that the lack of inhibitory transmission onto MNs in VGAT^-/- ^fetuses resulted in defects in the spontaneous and stimulus-induced movements *in vivo *despite the presence of excitatory synaptic transmission.

In addition to the defect in motor movement, trapezius muscle displayed atrophy in VGAT^-/- ^mice. Embryonic myogenesis progresses by the proliferation of myoblasts and fusion of myotubes, but it requires substantial cell death [32]. Physical forces play a significant role in the development and maintenance of skeletal muscle [33]. In cultured myoblasts, chronic and cyclic stretch results in an increase in cell death, including apoptosis [34]. Therefore, a possible explanation for the atrophy in VGAT^-/- ^trapezius muscle is that stretching of the trapezius muscle due to the hunched posture caused an increase in apoptosis during development.

### Phenotypes of VGAT and GAD67 knockouts outside of the brain

Ventral body wall closure abnormalities, such as omphalocele, are common human birth defects, but their molecular and cellular bases are poorly understood [35]. The mouse provides a model system to study the genetic defects and environmental insults that can lead to ventral body wall closure abnormalities [20]. In this study, omphalocele was observed in VGAT^-/- ^and GAD67^-/- ^mice, indicating that the lack of GABA signaling was involved in its onset. Omphalocele has been observed in K^+^-Cl^-^-cotransporter 2 (KCC2) knockout mice [36], and KCC2 is required for GABA- and/or glycine-induced hyperpolarizing responses [37]. In the KCC2 knockout mice, GABA and/or glycine signals continue to act in an excitatory, but not an inhibitory, manner. Therefore, the omphaloceles observed in both VGAT^-/- ^mice and GAD67^-/- ^mice resulted from defects in the inhibitory neurotransmission derived from the hyperpolarizing response. A lack of inhibitory transmission in VGAT^-/- ^mice may lead to motor deficits, such as a hunched posture. It is likely that the hunched posture resulted in increases in both intrathoracic and intraabdominal pressures and this increased pressure caused omphalocele.

Concerning the mechanism of onset of cleft palate, studies using knockout mice have revealed associations between cleft palate and mutation of genes related to GABA signaling, such as *GAD67 *and *GABRB3 *[8,23,38]. Because the lack of the GAD67 gene leads to a reduction in tongue movement [39], the sluggish tongue may be an obstacle to development of the palatal shelves.

The cleft palate and omphalocele phenotypes were more severe in VGAT^-/- ^mice than in GAD67^-/- ^mice. Glycinergic transmission is present in embryonic spinal cord and brainstem [40]. Hyperekplexia is a neurogenetic disorder caused mostly by mutations in the gene encoding the α1 subunit of glycine receptor and is characterized by an exaggerated startle response and neonatal hypertonia. In patients with hyperekplexia, the recurrent abdominal muscle contraction from the exaggerated startle response can increase the abdominal pressure and lead to omphalocele and inguinal hernia [41,42]. These reports suggest that a defect in glycinergic transmission is involved in the onset of omphalocele. A small amount of GABA is synthesized by another GAD isoform, GAD65, at the embryonic stage [8,43]. The differences in the severity between VGAT^-/- ^and GAD67^-/- ^mice must be due to the presence of both glycinergic and GAD65-produced GABAergic transmission in GAD67^-/- ^fetuses, but not in VGAT^-/- ^fetuses.

## Conclusion

In the present study, we established a VGAT knockout mouse, with which we demonstrated that VGAT is fundamental for GABAergic and/or glycinergic transmission. We also showed that VGAT is important for fetal growth and the development of muscle, liver and lung. The VGAT knockout mice described here may provide a useful tool for the study of specific functions of VGAT-dependent GABAergic and/or glycinergic transmission in mice. GABAergic neurons are classified into several subtypes according to the expression of chemical markers, such as parvalbumin and somatostatin [44,45]. Therefore, our floxed VGAT mice will be useful for conditional knockout studies to further investigate the role of VGAT in GABAergic neuronal subtypes.

## Methods

### Animals

All animal procedures were conducted in accordance with the guiding principles of the NIH under the review and approval of the Animal Care and Experimentation Committee, Gunma University, Showa Campus (Maebashi, Japan). Every effort was made to minimize the number of animals used and their suffering.

### Construction of the Targeting Vector

Genomic BAC clones containing the mouse VGAT (mVGAT) locus were purchased, and the regions covering the entire VGAT gene were subcloned [13]. A genomic fragment spanning exons 1-3 of the mVGAT gene was used for the targeting vector (Figure 1; targeting vector). The HindIII (in the 5'-flanking region) - KpnI (in the 3'-flanking region) fragment (7.5 kb) was subcloned into pBluescript II KS(-), and the 5'-loxP site was introduced into the XbaI site (in intron 1). The 5'-loxP site was flanked by a KpnI site artificially introduced for Southern blot analysis. The 7.5 kb fragment was used as the 5' homologous region containing the 5'-flanking region, exons 1-3 and the 3'-flanking region. The frt-flanked PGK-Neo cassette for positive selection of ES clones and the 3'-loxP site were inserted into the KpnI site (in the 3'-flanking region). The KpnI-BstEII fragment in the 3'-flanking region (3.5 kb) was added as the 3' homologous region. An MC1-DT-ApA cassette for negative selection [46] was ligated to the 3' end of the homologous region.

### Creation of a VGAT knockout allele

The linearized targeting vector was introduced by electroporation into ES cells (CCE) of 129/SvEv mouse origin, and G418-resistant colonies were screened by Southern blot analysis using probes outside of the targeting vector. KpnI-digested genomic DNA prepared from ES cell colonies was hybridized with 5' probes. The correctly targeted ES clones were injected into C57BL/6 blastocysts to produce germline chimeras. The germline chimeras were mated with C57BL/6 mice to establish the VGAT^floxneo/+ ^mouse line. VGAT^floxneo/+ ^mice were crossed with CAG-Cre mice [14] to excise exons 2 and 3 (VGAT knockout allele), and VGAT^+/- ^mice were obtained. We then intercrossed VGAT^+/- ^mice to generate VGAT^-/- ^mice. When we performed timed matings of the VGAT^+/- ^mice, the morning on the day of vaginal plug detection was designated E0.5.

Genotypes of VGAT^+/+^, VGAT^+/- ^and VGAT^-/- ^mice were determined by PCR using the following oligonucleotides: primer P1 (5'-AGTCTGATCCCGTGGCACTTCCAACTC-3') corresponding to intron 1 of the VGAT gene and primers P2 (5'-TCAGAGGCTTCTTCCTAGGGCTGCTG-3') and P3 (5'-GACCTCCCCCATTGCATAGAATGGCAC-3') corresponding to the 3'-flanking region of the VGAT gene. The primer set of P2 and P3 amplified a 183-bp fragment specific for the wild-type allele, and the primer set of P1 and P3 yielded a 430-bp fragment specific for the knockout allele.

### GAD67 knockout mice

We used homozygous GAD67-GFP (ℾneo) (GAD67^GFP/GFP^) mice as GAD67 knockout (GAD67^-/-^) mice. The generation of the GAD67-GFP (ℾneo) mice and their genotyping by PCR were described previously [47,48]. In the GAD67-GFP (ℾneo) mice, a cDNA encoding enhanced green fluorescent protein (EGFP) followed by an SV40 polyadenylation signal was targeted to the locus encoding GAD67 by homologous recombination, and the GAD67 gene was disrupted.

### Western blotting and measurement of neurotransmitter contents

For Western blotting, homogenates prepared from E18.5 mouse brain were separated by 7.5% SDS-polyacrylamide gel electrophoresis, transferred to nitrocellulose membrane (Whatman, Maidstone, UK), and probed with antibodies specific for VGAT (1:1000) [49], GAD65/67 (1:1000) [50], synaptophysin (1:1000) (Synaptic Systems, Goettingen, Germany), and β-actin (1:10000) (Abcam, Cambridge, UK). After the membranes were washed with Tris-HCl buffered saline containing 0.05% (w/v) Tween 20, the bound antibodies were visualized with horseradish peroxidase-conjugated goat anti-mouse IgG or anti-rabbit IgG (Jackson ImmunoResearch Laboratories, West Grove, PA) using the ECL Western blotting detection system (GE Healthcare, London, UK). Protein levels were quantified using Light Capture and its quantification software (ATTO, Tokyo, Japan). Expression levels were normalized to β-actin or synaptophysin levels, and the values are expressed as means ± SE. Statistical significance was assessed using Student's *t*-test.

GABA, glycine, and glutamate contents in the E18.5 mouse forebrain were measured according to previously described method [21,47].

### Electrophysiological recording in spinal cord

Embryos (E17.5-18.5) of control (VGAT^+/+^; n = 3, VGAT^+/-^; n = 5) and VGAT^-/- ^(n = 12) mice were decapitated and eviscerated, and the spinal cord was removed by ventral laminectomy. The isolated spinal cord was placed in a recording chamber perfused with oxygenated Ringer's solution (118.4 mM NaCl, 3 mM KCl, 2.52 mM CaCl_2_, 1.25 mM MgSO_4_, 25 mM NaHCO_3_, 1.18 mM KH_2_PO_4_, and 11.1 mM D-glucose aerated with 5% CO_2 _in O_2_) at room temperature. Recordings from MNs in the isolated spinal cord were performed as described previously [17]. Briefly, visually guided whole-cell tight-seal recording of MNs was performed with patch electrodes pulled from thick walled borosilicate glass to a final resistance of 5-8 MΩ. The electrode tips were filled with (in mM) 138 K-gluconate, 10 HEPES, 1 CaCl_2_, 5 ATP-Mg, and 0.3 GTP-Li. Intracellular signals were amplified with a Multiclamp 700B amplifier (Molecular Devices, Union City, CA), digitized at 5 kHz with the Digidata 1440A data acquisition system (Molecular Devices) and saved on a hard disk for off-line analysis. Electrical stimulations of lumbar ventral roots (VRs) were performed using glass suction electrodes. MNs were identified visually as cells with large soma in the ventral horn and by observing the antidromic firing activated by the electrical stimulation of the adjacent VR. All drugs (CNQX, AP5, strychnine and picrotoxin; Sigma-Aldrich, St. Louis, MO) were dissolved in Ringer's solution and bath-applied to the preparation. Analysis was performed using pClamp 10 software (Molecular Devices).

### Histology

VGAT^-/-^, VGAT^+/- ^and VGAT^+/+ ^mice at E18.5 were investigated. Samples were fixed in 10% (vol/vol) formaldehyde, dehydrated with a graded series of ethanol solutions, and embedded in paraffin. Three-micrometer sections were prepared, subjected to paraffin removal by immersion in xylene, rehydrated, and stained with hematoxylin-eosin. VGAT^+/- ^and VGAT^+/+ ^mice were mixed together and are referred to as control mice.

## Competing interests

The authors declare that they have no competing interests.

## Authors' contributions

Conceived and designed the experiments: KS, TK, HN, YN, MY, SK, AF, MF, KN, KO, YY. Performed the experiments: KS, TK, HN, TF, RH, ST, SE, MU, KI, MF, KN, KO, YY. Analyzed the data: KS, TK, HN, TF, RH, KI, AF, MF, KN, KO, YY. Contributed new reagents/analytical tools: ST, MM, JM. Wrote the paper: KS, TK, HN, AF, MF, KN, KO, YY. All authors read and approved the final manuscript.

## Supplementary Material

Additional file 1 **Supplementary Figure S1**. VGAT expression levels in VGAT mutant mice. (A, B) VGAT expression level was normal in adult VGAT^floxneo/floxneo ^mice. Western blot of whole brain homogenates from adult VGAT^+/+ ^(+/+) and VGAT^floxneo/floxneo ^(floxneo/floxneo) mice is shown (A). VGAT expression level normalized to β-actin was not different between VGAT^+/+ ^(+/+) and VGAT^floxneo/floxneo ^(floxneo/floxneo) mice (B). (C, D) VGAT expression level was reduced by about half in adult VGAT^+/- ^mice. Western blot of whole brain homogenates of adult VGAT^+/+ ^(+/+) and VGAT^+/- ^(+/-) mice is shown (C). VGAT expression level normalized to synaptophysin was significantly different between VGAT^+/+ ^(+/+) and VGAT^+/- ^(+/-) mice (D). Significance was tested by Student's t-test (*P < 0.05). Click here for file

## References

[B1] Roberts E Kuriyama K Biochemical-physiological correlations in studies of the gamma-aminobutyric acid system Brain Res 1968 8 1 35 10.1016/0006-8993(68)90170-4 4870412

[B2] McIntire SL Reimer RJ Schuske K Edwards RH Jorgensen EM Identification and characterization of the vesicular GABA transporter Nature 1997 389 870 876 10.1038/39908 9349821

[B3] Sagne C El Mestikawy S Isambert MF Hamon M Henry JP Giros B Gasnier B Cloning of a functional vesicular GABA and glycine transporter by screening of genome databases FEBS Lett 1997 417 177 183 10.1016/S0014-5793(97)01279-9 9395291

[B4] Bu DF Erlander MG Hitz BC Tillakaratne NJ Kaufman DL Wagner-McPherson CB Evans GA Tobin AJ Two human glutamate decarboxylases, 65-kDa GAD and 67-kDa GAD, are each encoded by a single gene Proc Natl Acad Sci USA 1992 89 2115 2119 10.1073/pnas.89.6.2115 1549570 PMC48607

[B5] Esclapez M Tillakaratne NJ Kaufman DL Tobin AJ Houser CR Comparative localization of two forms of glutamic acid decarboxylase and their mRNAs in rat brain supports the concept of functional differences between the forms J Neurosci 1994 14 1834 1855 8126575 10.1523/JNEUROSCI.14-03-01834.1994 PMC6577546

[B6] Stork O Ji FY Kaneko K Stork S Yoshinobu Y Moriya T Shibata S Obata K Postnatal development of a GABA deficit and disturbance of neural functions in mice lacking GAD65 Brain Res 2000 865 45 58 10.1016/S0006-8993(00)02206-X 10814732

[B7] Kubo K Nishikawa K Ishizeki J Hardy-Yamada M Yanagawa Y Saito S Thermal hyperalgesia via supraspinal mechanisms in mice lacking glutamate decarboxylase 65 J Pharmacol Exp Ther 2009 331 162 169 10.1124/jpet.109.156034 19571163

[B8] Asada H Kawamura Y Maruyama K Kume H Ding RG Kanbara N Kuzume H Sanbo M Yagi T Obata K Cleft palate and decreased brain gamma-aminobutyric acid in mice lacking the 67-kDa isoform of glutamic acid decarboxylase Proc Natl Acad Sci USA 1997 94 6496 6499 10.1073/pnas.94.12.6496 9177246 PMC21078

[B9] Dumoulin A Rostaing P Bedet C Lévi S Isambert MF Henry JP Triller A Gasnier B Presence of the vesicular inhibitory amino acid transporter in GABAergic and glycinergic synaptic terminal boutons J Cell Sci 1999 112 811 823 10036231 10.1242/jcs.112.6.811

[B10] Wojcik SM Katsurabayashi S Guillemin I Friauf E Rosenmund C Brose N Rhee JS A shared vesicular carrier allows synaptic corelease of GABA and glycine Neuron 2006 50 575 587 10.1016/j.neuron.2006.04.016 16701208

[B11] Aubrey KR Rossi FM Ruivo R Alboni S Bellenchi GC Le Goff A Gasnier B Supplisson S The transporters GlyT2 and VIAAT cooperate to determine the vesicular glycinergic phenotype J Neurosci 2007 27 6273 6281 10.1523/JNEUROSCI.1024-07.2007 17554001 PMC6672136

[B12] Saito K Nakamura K Kakizaki T Ebihara S Uematsu M Takamori S Yokoyama M Konishi S Mishina M Miyazaki JI Obata K Yanagawa Y Generation and analysis of vesicular GABA transporter knockout mouse [abstract] Neurosci Res 2006 55 S80

[B13] Ebihara S Obata K Yanagawa Y Mouse vesicular GABA transporter gene: genomic organization, transcriptional regulation and chromosomal localization Brain Res Mol Brain Res 2003 110 126 139 10.1016/S0169-328X(02)00648-4 12573541

[B14] Sakai K Miyazaki J A transgenic mouse line that retains Cre recombinase activity in mature oocytes irrespective of the cre transgene transmission Biochem Biophys Res Commun 1997 237 318 324 10.1006/bbrc.1997.7111 9268708

[B15] Wang YM Gainetdinov RR Fumagalli F Xu F Jones SR Bock CB Miller GW Wightman RM Caron MG Knockout of the vesicular monoamine transporter 2 gene results in neonatal death and supersensitivity to cocaine and amphetamine Neuron 1997 19 1285 1296 10.1016/S0896-6273(00)80419-5 9427251

[B16] de Castro BM De Jaeger X Martins-Silva C Lima RD Amaral E Menezes C Lima P Neves CM Pires RG Gould TW Welch I Kushmerick C Guatimosim C Izquierdo I Cammarota M Rylett RJ Gomez MV Caron MG Oppenheim RW Prado MA Prado VF The vesicular acetylcholine transporter is required for neuromuscular development and function Mol Cell Biol 2009 29 5238 5250 10.1128/MCB.00245-09 19635813 PMC2747982

[B17] Nishimaru H Restrepo CE Ryge J Yanagawa Y Kiehn O Mammalian motor neurons corelease glutamate and acetylcholine at central synapses Proc Natl Acad Sci USA 2005 102 5245 5249 10.1073/pnas.0501331102 15781854 PMC555035

[B18] Toribio RE Brown HA Novince CM Marlow B Hernon K Lanigan LG Hildreth BE Werbeck JL Shu ST Lorch G Carlton M Foley J Boyaka P McCauley LK Rosol TJ The midregion, nuclear localization sequence, and C terminus of PTHrP regulate skeletal development, hematopoiesis, and survival in mice FASEB J 2010 24 1947 1957 10.1096/fj.09-147033 20145205 PMC3140789

[B19] Yoda E Hachisu K Taketomi Y Yoshida K Nakamura M Ikeda K Taguchi R Nakatani Y Kuwata H Murakami M Kudo I Hara S Mitochondrial dysfunction and reduced prostaglandin synthesis in skeletal muscle of Group VIB Ca2+-independent phospholipase A2gamma-deficient mice J Lipid Res 2010 51 3003 3015 10.1194/jlr.M008060 20625036 PMC2936754

[B20] Brewer S Williams T Finally, a sense of closure? Animal models of human ventral body wall defects Bioessays 2004 26 1307 1321 10.1002/bies.20137 15551266

[B21] Fujii M Arata A Kanbara-Kume N Saito K Yanagawa Y Obata K Respiratory activity in brainstem of fetal mice lacking glutamate decarboxylase 65/67 and vesicular GABA transporter Neuroscience 2007 146 1044 1052 10.1016/j.neuroscience.2007.02.050 17418495

[B22] Baertschi S Zhuang L Trueb B Mice with a targeted disruption of the Fgfrl1 gene die at birth due to alterations in the diaphragm FEBS J 2007 274 6241 6253 10.1111/j.1742-4658.2007.06143.x 17986259

[B23] Condie BG Bain G Gottlieb DI Capecchi MR Cleft palate in mice with a targeted mutation in the gamma-aminobutyric acid-producing enzyme glutamic acid decarboxylase 67 Proc Natl Acad Sci USA 1997 94 11451 11455 10.1073/pnas.94.21.11451 9326630 PMC23502

[B24] McIntire SL Jorgensen E Horvitz HR Genes required for GABA function in Caenorhabditis elegans Nature 1993 364 334 337 10.1038/364334a0 8332190

[B25] Aragon C Lopez-Corcuera B Structure, function and regulation of glycine neurotransporters Eur J Pharmacol 2003 479 249 262 10.1016/j.ejphar.2003.08.074 14612155

[B26] Conti F Minelli A Melone M GABA transporters in the mammalian cerebral cortex: localization, development and pathological implications Brain Res Brain Res Rev 2004 45 196 212 10.1016/j.brainresrev.2004.03.003 15210304

[B27] Cherubini E Conti F Generating diversity at GABAergic synapses Trends Neurosci 2001 24 155 162 10.1016/S0166-2236(00)01724-0 11182455

[B28] Schousboe A Role of astrocytes in the maintenance and modulation of glutamatergic and GABAergic neurotransmission Neurochem Res 2003 28 347 352 10.1023/A:1022397704922 12608708

[B29] Sato K Yoshida S Fujiwara K Tada K Tohyama M Glycine cleavage system in astrocytes Brain Res 1991 567 64 70 10.1016/0006-8993(91)91436-5 1815830

[B30] Nishimaru H Iizuka M Ozaki S Kudo N Spontaneous motoneuronal activity mediated by glycine and GABA in the spinal cord of rat fetuses in vitro J Physiol 1996 497 131 143 8951717 10.1113/jphysiol.1996.sp021755 PMC1160918

[B31] Hanson MG Landmesser LT Characterization of the circuits that generate spontaneous episodes of activity in the early embryonic mouse spinal cord J Neurosci 2003 23 587 600 12533619 10.1523/JNEUROSCI.23-02-00587.2003 PMC6741864

[B32] Sandri M Carraro U Apoptosis of skeletal muscles during development and disease Int J Biochem Cell Biol 1999 31 1373 1390 10.1016/S1357-2725(99)00063-1 10641792

[B33] Proske U Morgan DL Muscle damage from eccentric exercise: mechanism, mechanical signs, adaptation and clinical applications J Physiol 2001 537 333 345 10.1111/j.1469-7793.2001.00333.x 11731568 PMC2278966

[B34] Liu J Liu J Mao J Yuan X Lin Z Li Y Caspase-3-mediated cyclic stretch-induced myoblast apoptosis via a Fas/FasL-independent signaling pathway during myogenesis J Cell Biochem 2009 107 834 844 10.1002/jcb.22182 19415683

[B35] Williams T Animal models of ventral body wall closure defects: a personal perspective on gastroschisis Am J Med Genet C Semin Med Genet 2008 148C 186 191 10.1002/ajmg.c.30179 18655100

[B36] Hubner CA Stein V Hermans-Borgmeyer I Meyer T Ballanyi K Jentsch TJ Disruption of KCC2 reveals an essential role of K-Cl cotransport already in early synaptic inhibition Neuron 2001 30 515 524 10.1016/S0896-6273(01)00297-5 11395011

[B37] Delpire E Cation-Chloride Cotransporters in Neuronal Communication News Physiol Sci 2000 15 309 312 11390932 10.1152/physiologyonline.2000.15.6.309

[B38] Homanics GE DeLorey TM Firestone LL Quinlan JJ Handforth A Harrison NL Krasowski MD Rick CE Korpi ER Makela R Brilliant MH Hagiwara N Ferguson C Snyder K Olsen RW Mice devoid of gamma-aminobutyrate type A receptor beta3 subunit have epilepsy, cleft palate, and hypersensitive behavior Proc Natl Acad Sci USA 1997 94 4143 4148 10.1073/pnas.94.8.4143 9108119 PMC20582

[B39] Tsunekawa N Arata A Obata K Development of spontaneous mouth/tongue movement and related neural activity, and their repression in fetal mice lacking glutamate decarboxylase 67 Eur J Neurosci 2005 21 173 178 10.1111/j.1460-9568.2004.03860.x 15654854

[B40] Greer JJ Funk GD Perinatal development of respiratory motoneurons Respir Physiol Neurobiol 2005 149 43 61 10.1016/j.resp.2005.03.017 15951250

[B41] Suhren O Bruyn GW Tuynman JA Hyperexplexia - A Hereditary Startle Syndrome J Neurol Sci 1966 3 577 605 10.1016/0022-510X(66)90047-5

[B42] Zhou L Chillag KL Nigro MA Hyperekplexia: a treatable neurogenetic disease Brain Dev 2002 24 669 674 10.1016/S0387-7604(02)00095-5 12427512

[B43] Ji F Kanbara N Obata K GABA and histogenesis in fetal and neonatal mouse brain lacking both the isoforms of glutamic acid decarboxylase Neurosci Res 1999 33 187 194 10.1016/S0168-0102(99)00011-5 10211762

[B44] Kawaguchi Y Kondo S Parvalbumin, somatostatin and cholecystokinin as chemical markers for specific GABAergic interneuron types in the rat frontal cortex J Neurocytol 2002 31 277 287 10.1023/A:1024126110356 12815247

[B45] Ascoli GA Alonso-Nanclares L Anderson SA Barrionuevo G Benavides-Piccione R Burkhalter A Buzsáki G Cauli B Defelipe J Fairén A Feldmeyer D Fishell G Fregnac Y Freund TF Gardner D Gardner EP Goldberg JH Helmstaedter M Hestrin S Karube F Kisvárday ZF Lambolez B Lewis DA Marin O Markram H Muñoz A Packer A Petersen CC Rockland KS Rossier J Rudy B Somogyi P Staiger JF Tamas G Thomson AM Toledo-Rodriguez M Wang Y West DC Yuste R Petilla terminology: nomenclature of features of GABAergic interneurons of the cerebral cortex Nat Rev Neurosci 2008 9 557 568 10.1038/nrn2402 18568015 PMC2868386

[B46] Yanagawa Y Kobayashi T Ohnishi M Kobayashi T Tamura S Tsuzuki T Sanbo M Yagi T Tashiro F Miyazaki J Enrichment and efficient screening of ES cells containing a targeted mutation: the use of DT-A gene with the polyadenylation signal as a negative selection maker Transgenic Res 1999 8 215 221 10.1023/A:1008914020843 10478491

[B47] Tamamaki N Yanagawa Y Tomioka R Miyazaki J Obata K Kaneko T Green fluorescent protein expression and colocalization with calretinin, parvalbumin, and somatostatin in the GAD67-GFP knock-in mouse J Comp Neurol 2003 467 60 79 10.1002/cne.10905 14574680

[B48] Kaneko K Tamamaki N Owada H Kakizaki T Kume N Totsuka M Yamamoto T Yawo H Yagi T Obata K Yanagawa Y Noradrenergic excitation of a subpopulation of GABAergic cells in the basolateral amygdala via both activation of nonselective cationic conductance and suppression of resting K+ conductance: a study using glutamate decarboxylase 67-green fluorescent protein knock-in mice Neuroscience 2008 157 781 797 10.1016/j.neuroscience.2008.09.029 18950687

[B49] Takamori S Riedel D Jahn R Immunoisolation of GABA-specific synaptic vesicles defines a functionally distinct subset of synaptic vesicles J Neurosci 2000 20 4904 4911 10864948 10.1523/JNEUROSCI.20-13-04904.2000 PMC6772304

[B50] Asada H Kawamura Y Maruyama K Kume H Ding R Ji FY Kanbara N Kuzume H Sanbo M Yagi T Obata K Mice lacking the 65 kDa isoform of glutamic acid decarboxylase (GAD65) maintain normal levels of GAD67 and GABA in their brains but are susceptible to seizures Biochem Biophys Res Commun 1996 229 891 895 10.1006/bbrc.1996.1898 8954991

