# Supplementary figures and images for: The physiological roles of vesicular GABA transporter during embryonic development: a study using knockout mice
Source: Mol Brain. 2010 Dec 30;3:40. doi: 10.1186/1756-6606-3-40 (PMC3023674; doi:10.1186/1756-6606-3-40)

**A**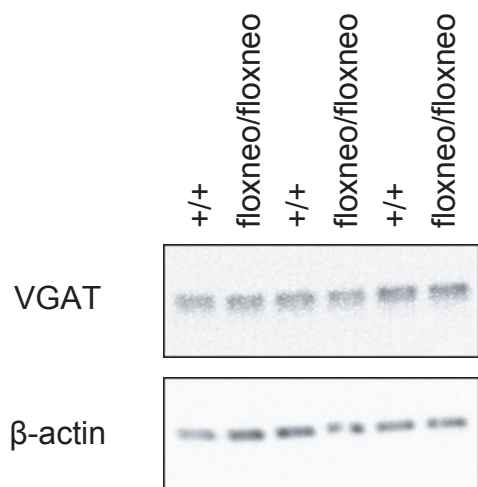**B**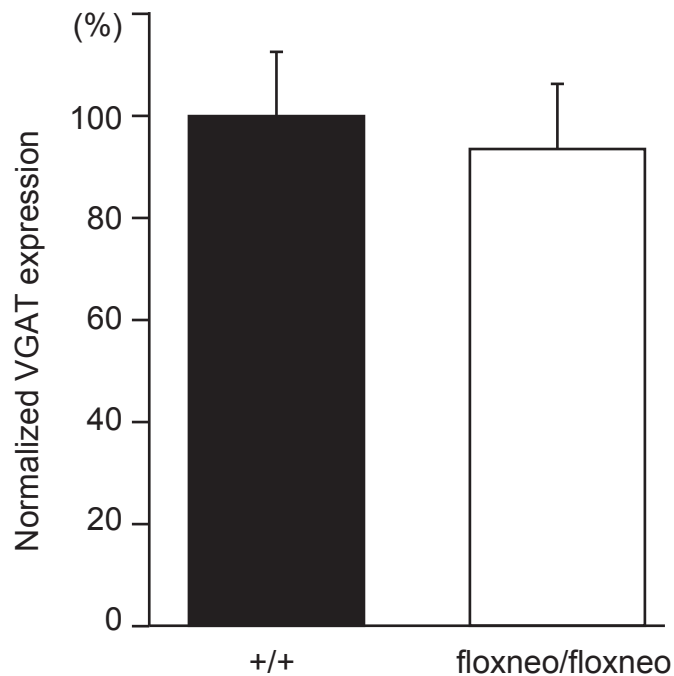**C**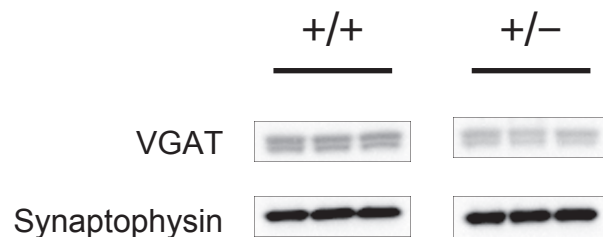**D**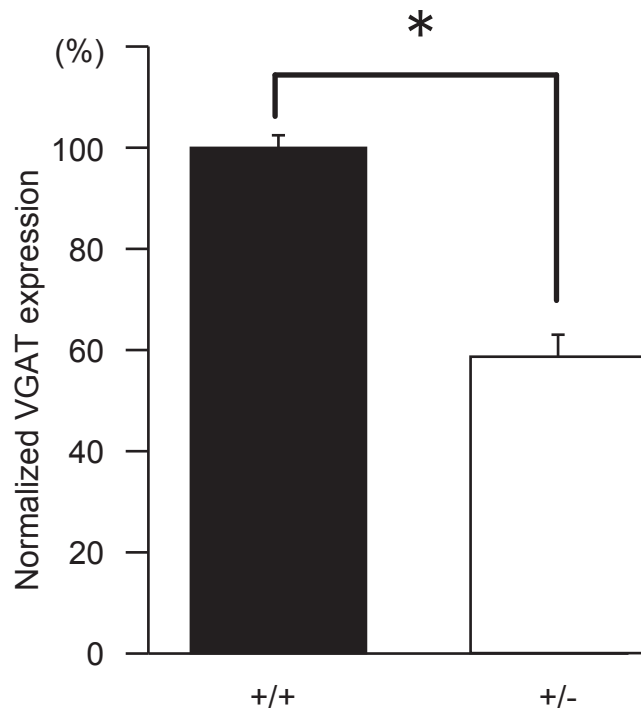

Supplement: Additional file 1 — Supplementary Figure S1. VGAT expression levels in VGAT mutant mice. (A, B) VGAT expression level was normal in adult VGATfloxneo/floxneo mice. Western blot of whole brain homogenates from adult VGAT+/+ (+/+) and VGATfloxneo/floxneo (floxneo/floxneo) mice is shown (A). VGAT expression level normalized to β-actin was not different between VGAT+/+ (+/+) and VGATfloxneo/floxneo (floxneo/floxneo) mice (B). (C, D) VGAT expression level was reduced by about half in adult VGAT+/- mice. Western blot of whole brain homogenates of adult VGAT+/+ (+/+) and VGAT+/- (+/-) mice is shown (C). VGAT expression level normalized to synaptophysin was significantly different between VGAT+/+ (+/+) and VGAT+/- (+/-) mice (D). Significance was tested by Student's t-test (*P < 0.05). [file 1756-6606-3-40-S1.PDF]
